# Early biochemical outcomes following neoadjuvant/adjuvant relugolix with stereotactic body radiation therapy for intermediate to high risk prostate cancer

**DOI:** 10.3389/fonc.2023.1289249

**Published:** 2023-10-17

**Authors:** Lindsey Gallagher, Jerry Xiao, Jessica Hsueh, Sarthak Shah, Malika Danner, Alan Zwart, Marilyn Ayoob, Thomas Yung, Tiffany Simpson, Mark Fallick, Deepak Kumar, Paul Leger, Nancy A. Dawson, Simeng Suy, Sean P. Collins

**Affiliations:** ^1^ Department of Radiation Medicine, MedStar Georgetown University Hospital, Washington, DC, United States; ^2^ Department of Oncology, Lombardi Comprehensive Cancer Center, Georgetown University Medical Center, Washington, DC, United States; ^3^ Medical Science Department, Myovant Sciences, Inc, United States; ^4^ Biotechnology Research Institute, North Carolina Central University, Durham, NC, United States

**Keywords:** prostate adenocarcinoma, relugolix, stereotactic body radiation therapy (SBRT), androgen deprivation therapy (ADT), testosterone suppression

## Abstract

**Introduction:**

Injectable GnRH receptor agonists have been shown to improve cancer control when combined with radiotherapy. Prostate SBRT offers an abbreviated treatment course with comparable efficacy to conventionally fractionated radiotherapy. Relugolix is a new oral GnRH receptor antagonist which achieves rapid, sustained testosterone suppression. This prospective study sought to evaluate early testosterone suppression and PSA response following relugolix and SBRT for intermediate to high prostate cancer.

**Methods:**

Relugolix was initiated at least 2 months prior to SBRT. Interventions to improve adherence were not utilized. PSA and total testosterone levels were obtained prior to and 1-4 months post SBRT. Profound castration was defined as serum testosterone ≤ 20 ng/dL. Early PSA nadir was defined as the lowest PSA value within 4 months of completion of SBRT. Per prior trials, we examined the percentage of patients who achieved PSA level of ≤ 0.5 ng/mL and ≤ 0.2 ng/mL during the first 4 months post SBRT.

**Results:**

Between July 2021 and January 2023, 52 men were treated at Georgetown with relugolix (4-6 months) and SBRT (36.25-40 Gy in 5 fractions) per an institutional protocol (IRB 12-1775). Median age was 71 years. 26.9% of patients were African American and 28.8% were obese (BMI ≥30 kg/m2). The median pretreatment PSA was 9.1 ng/ml. 67% of patients were ≥ Grade Group 3. 44 patients were intermediate- and 8 were high-risk. Patients initiated relugolix at a median of 3.6 months prior to SBRT with a median duration of 6.2 total months. 92.3% of patients achieved profound castration during relugolix treatment. Poor drug adherence was observed in 2 patients. A third patient chose to discontinue relugolix due to side effects. By post-SBRT month 4, 87.2% and 74.4% of patients achieved PSA levels ≤ 0.5 ng/ml and ≤ 0.2 ng/ml, respectively.

**Discussion:**

Relugolix combined with SBRT allows for high rates of profound castration with low early PSA nadirs. We observed a 96% testosterone suppresion rate without the utilization of scheduled cues/reminders. This finding supports the notion that patients with localized prostate cancer can consistently and successfully follow an oral ADT protocol without daily reminders. Given relugolix’s potential benefits over injectable GnRH receptor agonists, its usage may be preferred in specific patient populations (fear of needles, prior cardiovascular events). Future studies should focus on boundaries to adherence in specific underserved populations.

## 1 Introduction

In 2022, there was an estimated 268,490 new cases of prostate cancer in the United States (1). Prostate cancer continues to be the leading cause of new cancer diagnoses, comprising 11% of all male cancer-related deaths in 2022 (1). For intermediate to high risk prostate cancer, the National Comprehensive Cancer Network (NCCN) guidelines endorse radiation therapy (RT) plus ADT (2). ADT in conjunction with conventionally fractionated radiation therapy significantly improves metastases-free and overall survival (3). Radiation dose escalation does not improve either of these important endpoints (4). However previous work has shown that SBRT with radiobiological dose escalation can achieve high rates of cancer control in unfavorable prostate cancer with minimal toxicity (5). As with external beam radiation therapy (EBRT), early data suggests that the addition of ADT to SBRT for high to intermediate risk prostate cancer may also reduce local persistence of disease and biochemical recurrence (6, 7). Unfortunately, ADT combined with RT remains underutilized possibly due to bothersome persistent side effects and its potentially negative impact on cardiovascular comorbidities (8).

In recent decades, the potential benefits of GnRH antagonists have been evaluated. Degarelix was the first readily available GnRH antagonist, exclusively offered in an injectable formulation (9). Although it is strongly efficacious in achieving testosterone suppression, Degarelix is associated with a high frequency of painful, injection site hypersensitivity reactions compared to GnRH agonist Leuprolide (40% versus <1%) (9). Approved by the FDA in 2020, relugolix is an oral ADT that suppresses gonadotropic release from the pituitary gland, thus decreasing concentrations of testosterone (10). The HERO study investigated the efficacy of this oral GnRH receptor antagonist compared to GnRH agonist Leuprolide. The randomized Phase 3 trial demonstrated the superiority of relugolix in achieving and maintaining castration, as well as a quicker testosterone recovery following discontinuation (11). On day 4 of use, 56.0% of patients receiving relugolix reached castrate level versus 0% with Leuprolide (11). Men treated with relugolix also maintained castration through 48 weeks at a rate of 96.7% compared to 88.8% with Leuprolide (11). These patients had a 54% lower risk of major adverse cardiovascular (CV) event after 12 months (11). The incidence of major adverse CV events favored relugolix at 2.9% versus 6.2% in the leuprolide group especially given similar distribution of CV risk factors between the two treatment arms (11). Importantly, the study exhibited a 99% adherence rate with oral relugolix using daily audible reminders (11).

Neoadjuvant/adjuvant relugolix (6 months) has been studied in intermediate to high risk prostate cancer in combination with conventionally fractionated radiation therapy (79.2 Gy in 44 fractions) (12). With relugolix, 95% achieved castration (total testosterone < 50 ng/dL, 1.73 nmol/L) and 82% reached profound castration (total testosterone < 20 ng/dL; 0.7 nmol/L). As with the HERO study, interventions to improve adherence were utilized (12). While oral ADTs have potential advantages, their real-world effectiveness is dependent on patient adherence (13). Bothersome side effects such as hot flashes, fatigue and decreased libido may lead to drug holidays and/or early cessation (14). This may be a bigger problem in minority and other underserved populations (15). In addition, patient characteristics such as obesity and unrecognized drug interactions could limit its relugolix real world effectiveness (13). Our investigation sought to evaluate real world early testosterone suppression and PSA response following relugolix and SBRT for intermediate to high risk prostate cancer.

## 2 Materials and methods

We conducted an IRB approved, prospective study (IRB 12-1175) of men with intermediate to high risk prostate cancer treated at MedStar Georgetown University Hospital. Patients were treated per institutional protocol with short-term relugolix (4-6 months) and SBRT (36.25-40 Gy in 5 fractions). Risk groups were defined using the D’Amico criteria. Other patient and treatment characteristics such as age, race, BMI, prostate volume, pretreatment PSA, T stage, Gleason score, and dose were acquired from the medical records.

### 2.1 Drug treatment

Neoadjuvant relugolix was initiated at least 2 months prior to SBRT with loading dose of 360 mg on the first day and continue treatment with a 120 mg dose taken orally once daily at approximately the same time each day. For patients with favorable intermediate risk disease, the decision to prescribe relugolix was made based on Decipher test results.

### 2.2 SBRT treatment planning and delivery

SBRT was delivered using the CyberKnife robotic radiosurgical system (Accuray Inc., Sunnyvale, CA) as previously described (16). Plans were inhomogeneous by design however prescription dose was prescribed to the 83% isodose line. Approximately two months following the initiation of relugolix, gold fiducials were transperineally placed into the prostate. One week after fiducial placement, CT and high-resolution MR images were obtained for treatment planning. The clinical target volume (CTV) included the prostate and proximal seminal vesicles. The planning target volume (PTV) included a uniform 3 mm expansion around the CTV. In general, each patient initiated treatment 2-4 weeks following treatment simulation. The prescription dose to the PTV was 36.25-40 Gy delivered in 5 fractions of 7.25-8 Gy over 1-2 weeks. Care was taken to avoid treatment beams that directly traversed the testes, and the testicular scatter dose was limited (D20% < 2 Gy).

### 2.3 Follow up and assessment

Early PSA and total testosterone levels were collected at two timepoints: immediately prior to SBRT initiation and 1-4 months after SBRT. We defined both effective and profound castration as a serum testosterone of ¾50 ng/dL (¾1.73 nmol/L) and ¾ 20 ng/dL (¾0.7 nmol/L) respectively as previously described (7). Early PSA nadir was demarcated as the lowest PSA value within 4 months of SBRT completion. We assessed the proportion of patients who achieved early PSA nadirs ≤ 1.0, ≤ 0.5, ≤ 0.2, ≤ 0.1, and <0.1 ng/mL at each of three timepoints: at SBRT, 1-4 months post-SBRT, and 5-8 months post-SBRT. In line with the efficacy endpoints defined in prior trials, we determined the percentage of patients reaching early PSA nadirs ≤ 0.5 ng/mL and ¾ 0.2 ng/mL during the first 4 months post SBRT (17, 18). Poor drug adherence was characterized as failure to reach profound castration, or testosterone ¾ 20 ng/dL, at any time point. Figures were obtained using R programming.

## 3 Results

### 3.1 Patient, treatment, and tumor characteristics

Patient, treatment, and tumor characteristics are described in **Table 1**. Between July 2021 and January 2023, 52 men with intermediate to high risk prostate cancer were treated at Georgetown University Hospital. Patients ranged from 49 to 88 years old, with a median age of 71. 62% of cohort were Caucasian and 27% African American. The majority (48%) of men were characterized as overweight (BMI between 25 and 29.9 kg/m^2^) while 29% fell within the obese category (BMI ≥30 kg/m^2^). Median pretreatment prostate specific antigen (PSA) was 8.1 ng/ml and ranged from 3.6 up to 39.3 ng/ml. 67% of patients were ≥ Grade Group 3. 44 patients were classified as intermediate- and 8 were high-risk. Of the intermediate group, patients were predominantly diagnosed with unfavorable disease. Patients initiated relugolix at a median of 3.6 months (range 2.6-5.7 months) prior to SBRT with a median duration of 6.2 total months (range 3.9-9.1 months).

**Table 1:** Patient, tumor, and treatment characteristics.

	n=52
Age (years)	
Mean	71
Median (range)	72 (49-88)
Race	
Caucasian	32 (61.5%)
African American	14 (26.9%)
Other	6 (11.5%)
BMI (kg/m^2^)	
<18.5	0 (0%)
18.5-24.9	12 (23.1%)
25-29.9	25 (48.1%)
30-34.9	8 (15.4%)
35-39.9	5 (9.6%)
40-44.9	2 (3.8%)
Pretreatment PSA (ng/ml)	
Mean	9.1
Median	8.1 (3.6-39.3)
Gleason Score	
6 (3 + 3)	4 (7.7%)
7 (3 + 4)	13 (25.0%)
7 (4 + 3)	28 (53.8%)
8 (4 + 4), 9 (4 + 5)	7 (13.5%)
Risk Group	
Intermediate - Favorable	12 (23.1%)
Intermediate - Unfavorable	32 (61.5%)
High	8 (15.4%)
Prostate volume (cc)	
Mean	43.1
Median	40 (15-124)
% of total cores involved	
Mean	43.6
Median	38.0 (7.7-100)
Maximum % of a single involved core	
Mean	57.0
Median	60 (5-95)
SBRT Dose (Gy)	
36.25	48 (92.3%)
40, 36.25	4 (7.7%)

### 3.2 Total testosterone levels

**Figure 1:**
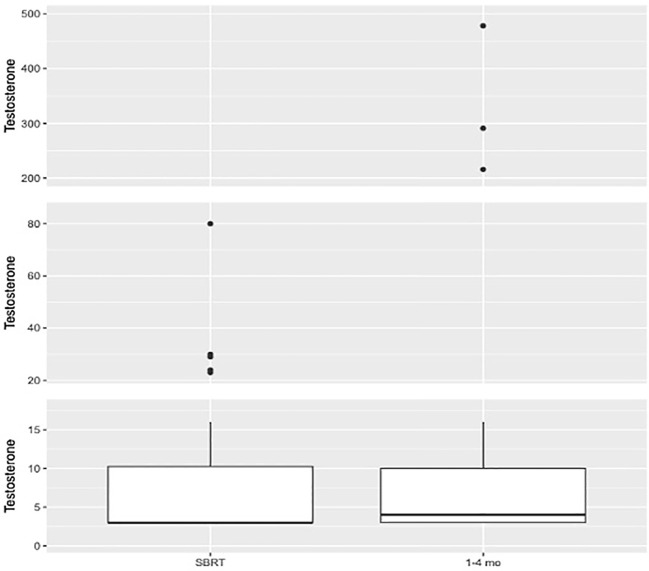
Box plot illustrating distribution of testosterone values (ng/dl) at SBRT (N=52. Mean=8.38. Median=3.0. Q1 = 3.0. Q3 = 10.25) and 1–4-month interval (N=39. Mean=31.05. Median=4.0. Q1 = 3.0. Q3 = 10.0).

See **Table 2** for summary of testosterone responses following neoadjuvant relugolix. At the time of SBRT, 98.1% of patients achieved a testosterone level ≤50 ng/dl (effective castration) while 90.4% reached testosterone ≤20 ng/dl (profound castration). Following SBRT (1-4 months), 92.3% of patients reached profound castration. 48.7% achieved testosterone ≤3 ng/dl. Poor drug adherence was observed in two patients. At 1-4 months post SBRT, both patients fell within normal testosterone values of 291 and 478 ng/dl (**Figure 1**). A third patient chose to discontinue relugolix shortly after SBRT due to side effects, reaching a normal testosterone of 216 ng/dl within three months post-SBRT (**Figure 1**).

**Table 2:** Percentage of patients reaching given testosterone level in months following relugolix + SBRT treatment.

Testosterone (ng/dl)	At SBRT	1-4 (months)
≤50	51 (98.1%)	36 (92.3%)
≤20	47 (90.4%)	36 (92.3%)
≤3	29 (67.6%)	19 (48.7%)
N	52	39

### 3.3 Early PSA nadirs

**Figure 2:**
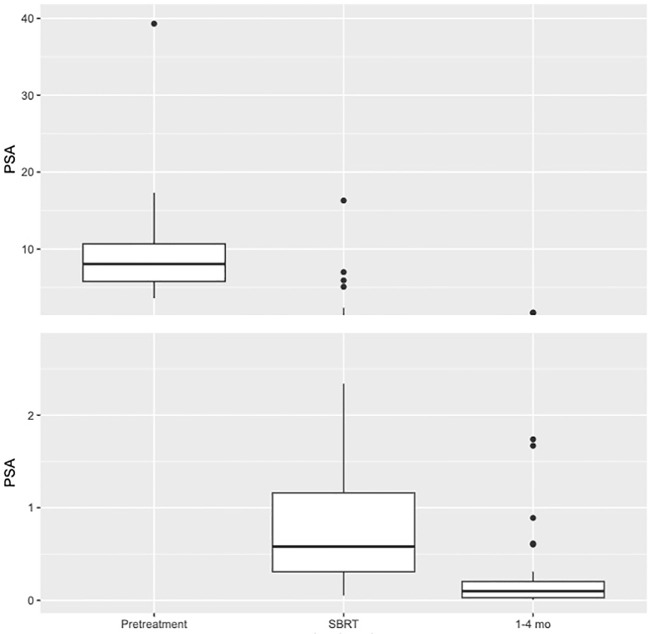
Box plot illustrating distribution of PSA values (ng/ml) at Pretreatment (N=52. Mean=9.09. Median=8.05. Q1 = 5.78. Q3 = 10.68), SBRT (N=52. Mean=1.29. Median=0.58. Q1 = 0.31. Q3 = 1.16), and 1–4-month interval (N=39. Mean=0.22. Median=0.1. Q1 = 0.03. Q3 = 0.2).

See **Table 3** for summary of early PSA nadir responses following neoadjuvant relugolix. Patients achieved a median PSA level of 0.58 ng/ml at the time of SBRT and 0.1 ng/ml 1-4 months after SBRT. At SBRT initiation, 71.2% of patients reached a PSA level ≤ 1.0 ng/ml. At this same time point, 46.2% and 13.5% of patients achieved PSA levels ≤ 0.5 ng/ml and ≤ 0.2 ng/ml, respectively. By 1-4 months post-SBRT, 94.9% reached early PSA nadir ≤ 1.0 ng/ml. 87.2% and 74.4% of men achieved PSA levels ≤ 0.5 ng/ml and ≤ 0.2 ng/ml respectively during the first 4 months following SBRT (**Figure 2**). We performed subgroup analyses of favorable intermediate, unfavorable intermediate, and high risk patients and found no significant differences in early PSA nadirs between disease risk groups.

**Table 3:** Percentage of patients reaching given PSA level in months following relugolix + SBRT treatment.

PSA (ng/ml)	@ SBRT	1-4 (months)	5-8
≤1	37 (71.2%)	37 (94.9%)	14 (93.3%)
≤0.5	24 (46.2%)	34 (87.2%)	11 (73.3%)
≤0.2	7 (13.5%)	29 (74.4%)	7 (46.7%)
≤0.1	2 (3.8%)	24 (61.5%)	4 (26.7%)
<0.1	2 (3.8%)	19 (48.7%)	3 (20.0%)
N	52	39	15

## 4 Discussion

Injectable GnRH agonists remain the most common choice for ADT in men with intermediate to high prostate cancer. However, patients face a variety of challenges and complications while undergoing treatment with these agents. First, they require regular injections that are inconvenient and painful. In addition, a clinically significant amount of patients report a fear of needles (19). Eek et al. found that oncology patients generally prefer oral over intravenous/injectable therapy due to convenience, increased efficacy perception, and prior experiences (20). Further difficulties present with GnRH agonists secondary to their intrinsic activating activity. Upon initiation of therapy, patients experience an early testosterone surge. This testosterone flare can lead to urinary retention/obstruction (9). Patients may also experience significant delays to castration (21). Likewise, testosterone recovery following discontinuation of GnRH agonists can be characterized as prolonged, uncontrolled, and unpredictable (22). An investigation of 307 prostate cancer patients by Nascimento et al. demonstrated that 24% of their cohort never regained normal testosterone values (>300 ng/dL) within 2 years of ADT cessation (23). Cardiovascular (CV) disease is currently recognized as the leading source of morbidity in males with prostate cancer, representing 27-34% of all-cause deaths (24). GnRH agonists have been long known to increase the risk of CV events particularly stroke and myocardial infarction (6). ADT with oral GnRH antagonist Relugolix may help ease these challenges and reduce the risk of serious complications.

Numerous studies have shown that PSA response to neoadjuvant ADT predicts long-term cancer control (25–29). In our study, greater than 90% of men achieved profound castration by the initiation of SBRT. This is comparable to previously reported studies of relugolix that utilized interventions to improve adherence (11, 12). With this high rate of profound castration in is not surprising that 71.2% and 46.2% achieved pre-SBRT PSA nadirs of ¾ 1 ng/ml and ¾ 0.5 ng/ml, respectively. This compares favorably with previous results utilizing 2-9 months neoadjuvant injectable GnRH agonists (¾ 1 ng/ml = 56%) (25). In this study, a post-neoadjuvant ADT PSA nadir of ¾ 1 ng/ml predicted a higher rate of biochemical control and a higher overall survival in men with unfavorable prostate cancer treated with EBRT (25). In a similar study, utilizing 3 months neoadjuvant injectable GnRH agonist with concurrent flutamide achieved an early PSA nadir of ¾ 0.5 ng/ml in 21.8% of men (26). Men with a post-neoadjuvant ADT PSA nadir of ¾ 0.5 ng/ml experienced a 5-year PSA relapse-free survival rate of 74%, as compared with 40% for patients with a higher PSA nadir (26). The etiology of these improved early PSA nadirs prior to SBRT is unknown but could be related to increases in sustained profound castration with relugolix (11).

Although guidelines define castrate testosterone levels of <50 ng/dL, there is evidence suggesting that serum testosterone levels of <20 ng/dL may improve clinical outcomes in certain clinical situations (30). For example, Klotz et al. ascertained that lower levels of testosterone during ADT (<0.7 nmol/L) in patients with biochemical recurrence correlated with an increase in cause-specific survival (CSS) (31). Still, it remains unclear how testosterone levels of <20 ng/dL affect the radio sensitizing ability of ADT.

The early PSA nadir at the end of therapy (RT and ADT) has been shown to be a surrogate endpoint for prostate cancer specific mortality (18, 32, 33). DFCI 95-096 first established an overall survival advantage for short term GnRH agonist therapy (6 months) plus RT (70 Gy) for intermediate risk disease (34). Likewise, TROG 96.01 showed an improved all-cause mortality for short term GnRH agonist therapy (3-6 months)/flutamide plus RT (66 Gy) (35). 95% and 75% achieved an early PSA nadir ≤ 0.5 ng/ml at the end of therapy in the DFCI 95-096 and TROG 96.01 studies respectively (32). In our study, 87.2% of patients 1-4 months post SBRT achieving a PSA level ≤ 0.5 ng/ml. Given its reported prognostic value, Kaplan et al. used a PSA cutoff level ≤ 0.2 ng/ml in an open-label, phase 2 trial that assessed the effectiveness of Enzalutamide and external beam RT for intermediate risk prostate cancer (17, 32). It was hypothesized that 60% of patients would achieve PSA ≤ 0.2 ng/ml, with 77% of patients ultimately meeting the endpoint (17). Similarly in our study, 74.4% of patients reached early PSA nadirs ≤ 0.2 ng/ml 1-4 months after SBRT. The difference between early PSA nadirs between studies is likely multifactorial including variable grade/volume disease, length of ADT, follow-up timepoints and sporadic non-adherence.

Participants of the HERO trial received special pill bottles with audible reminders which likely improved compliance rates and ensured continued testosterone suppression (36). Given that such interactive containers are expensive and not commercially available to the average patient, we did not utilize any scheduled cues as part of our study. Nonetheless, we observed an excellent compliance rate of 96% without prompting. Relevantly, 38% of our study population was non-Caucasian and represented various socioeconomic and racial backgrounds (37). The high compliance rate seen with a socially diverse patient cohort further validates that patients can consistently and successfully follow an oral ADT protocol. It is also key to mention the average effective and elimination half-lives (t_1/2_) of relugolix are approximately 25 hours and 36-65 hours respectively, indicating that testosterone levels are unlikely to be impacted by a single missed dose (38, 39). Importantly, 97% and 86% of men remained at castrate levels upon temporary interruption of treatment for 7 and 14 days, respectively (39).

Of note, poor drug adherence was observed in two of our study participants. Both patients were non-English speaking which may have contributed to adherence difficulties. Such findings emphasize the need to consider patients’ functional status and level of support when planning for ADT especially with relugolix. In select patient populations, we propose the use of regular testosterone checks to monitor adherence. Given the negative impact of possible noncompliance, future research should focus on obstacles to medication adherence. In addition, one patient voluntarily discontinued relugolix prior to completion of treatment due to side effects. Shucheng et al. highlighted the diversity of needs in individuals with prostate cancer and importance of patient empowerment (40). We utilized a shared decision-making model and encouraged patient involvement throughout treatment. This one patient’s testosterone levels quickly returned to normal range within weeks of stopping relugolix. The cessation responses align with the swift testosterone recoveries noted in the HERO study.

Limitations of our investigation are secondary to its small size and minor variations in treatment scheduling. Although we aimed to treat all patients for a total of 4-6 months with initiation 2 months prior to SBRT, there was heterogeneity in the timing of relugolix therapy. We did not examine whether there were any differences in outcomes depending on the timeliness of relugolix schedule parameters.

## 5 Conclusions

This study supports the use of relugolix and SBRT for the treatment of intermediate to high risk prostate cancer. High rates of profound castration and low early PSA nadirs were observed through combination treatment with relugolix and SBRT. With the known advantages of relugolix over injectable GnRH receptor agonists, its usage may be preferred especially in patients with a fear of needles or history of prior cardiovascular events. Further follow up relating to medication compliance and cost are needed to address potential real-world barriers. Patient reported quality of life outcomes on relugolix are also an active area of investigation.

## Data availability statement

The raw data supporting the conclusions of this article will be made available by the authors, without undue reservation.

## Ethics statement

The studies involving humans were approved by Georgetown-MedStar IRB Systemic. The studies were conducted in accordance with the local legislation and institutional requirements. The participants provided their written informed consent to participate in this study.

## Author contributions

LG: Conceptualization, Data curation, Formal Analysis, Investigation, Methodology, Validation, Writing – original draft, Writing – review & editing. JX: Formal Analysis, Visualization, Writing – review & editing. JH: Data curation, Writing – review & editing. SSh: Writing – review & editing. MD: Conceptualization, Data curation, Writing – review & editing. AZ: Conceptualization, Data curation, Resources, Writing – review & editing. MA: Resources, Writing – review & editing. TY: Conceptualization, Data curation, Writing – review & editing. TS: Data curation, Writing – review & editing. MF: Conceptualization, Formal Analysis, Investigation, Project administration, Writing – review & editing. DK: Data curation, Investigation, Methodology, Writing – review & editing. PL: Conceptualization, Data curation, Writing – review & editing. ND: Conceptualization, Data curation, Investigation, Methodology, Writing – review & editing. SSu: Conceptualization, Data curation, Investigation, Methodology, Project administration, Writing – review & editing. SC: Conceptualization, Data curation, Formal Analysis, Funding acquisition, Investigation, Methodology, Resources, Supervision, Validation, Writing – original draft, Writing – review & editing.
